# Knowledge About Alzheimer’s Disease in Saudi Arabia

**DOI:** 10.7759/cureus.50188

**Published:** 2023-12-08

**Authors:** Fahi El-Gamal, Ali K Alali, Muaadh H Mashaabi, Abdulhakim A Alsaghah, Faisal A Shukr, Syed Y Kadri

**Affiliations:** 1 Family Medicine, Ibn Sina National College, Jeddah, SAU; 2 Medical School, Ibn Sina National College, Jeddah, SAU

**Keywords:** saudi arabia, alzheimer’s disease, early-onset alzheimer’s disease, alzheimer care, alzheimer’s dementia

## Abstract

Background

Alzheimer’s disease (AD) is the most common neurodegenerative dementia which constitutes a major healthcare problem globally. This study aimed to assess the pattern and determinants of knowledge about AD in Saudi Arabia.

Methodology

In this cross-sectional study, information was collected from 286 participants using a validated questionnaire on seven subdomains of knowledge about AD. SPSS (IBM Corp., Armonk, NY, USA) was used for data analysis. Different tests of significance such as the chi-square test and principal component factor analyses were employed. The level of significance was set at 0.05.

Results

The 25 questions on knowledge, attitudes, and practice about AD had a high Cronbach’s alpha (0.911). All scores on the seven subdomains of knowledge, attitudes, and practice about AD were significantly correlated with each other (p < 0.05). The knowledge of the population about AD was very low, with the majority of the correct responses for questions on the different subdomains of knowledge about AD ranging from 10% to 67% (76% of the answers were below 50% correct answers). Females and smokers had higher scores on most subdomains compared to males and non-smokers (p < 0.05).

Conclusions

Knowledge of the population about AD is very low. As the subdomains of knowledge on AD are correlated, healthcare professionals should implement health education programs on AD to increase the knowledge of the population regarding the course, symptoms, risk factors, treatment and management, and caregiving and reveal their association with the impact of AD on the health of affected individuals.

## Introduction

Alzheimer’s disease (AD), the most common neurodegenerative dementia, is characterized by the formation of amyloid plaques in the brain, loss of neuronal tissue, and the appearance of neurofibrillary tangles inside neurons, with subsequent brain atrophy, ongoing neurodegeneration, and cognitive impairment [1,2]. AD is significantly associated with increased age and family history and may occur due to traumatic brain damage [3-7].

Disorientation, cognitive decline, generalized confusion, and memory impairment are the main clinical symptoms of AD [8-10]. Individuals with AD may show warning symptoms such as sleep disturbance, mood changes, personality changes, and language difficulties [11,12]. AD occurs more commonly in the elderly [5]. Pathological lesions are usually preceded by oxidative stress [13,14]. AD is significantly more common in individuals whose jobs entail lower mental demands and higher physical demands [15,16]. Chronic diseases associated with the incidence and worsening of AD include diabetes mellitus, renal disease, cardiovascular disease, and high cholesterol [17]. Depression is also associated with the occurrence of AD, with both adversely affecting the quality of life of the patients [18]. Dementia is a prevailing neurodegenerative disease globally, with an incidence of 6.4% in Saudi Arabia [19]. Women after the ages of 80-85 years are at a greater risk of AD compared to men. This increased risk is related to the depletion of endogenous estrogen in the menopausal period [14]. Cigarette smoking increases the risk of AD [17,18]. This study aimed to explore the association of different sociodemographic characteristics with the knowledge about AD in a population from Saudi Arabia.

## Materials and methods

This cross-sectional study employed a non-probability convenient sampling technique. Data were collected via online Google Forms distributed to individuals from Saudi Arabia. The sample size was determined using G*power software using an α of 0.05, power of 0.95, effect size of 0.3, and degree of freedom of 5. The minimal sample size required was 277. All 286 individuals who received the form, filled it, and sent it back were included in this study. For data collection, a predesigned questionnaire was used which included information on the sociodemographic and clinical characteristics of the participants. The validated Polish Alzheimer’s Disease Knowledge Scale (ADKS) questionnaire was used [19] to describe seven subdomains, namely, risk factors; evaluation and diagnosis; symptoms; course; life effect, caregiving; and therapy and management of AD (Appendices). Each question was given three codes as follows: 3 for true, 2 for false, and 1 for do not know. The score for each domain was calculated using a simple additive method [20,21].

Statistical analysis

Data were analyzed using the SPSS software (IBM Corp., Armonk, NY, USA). The chi-square test of significance, Student’s t-test, and analysis of variance were used. The multifactorial principal component factor analysis was employed, and variables with weights of 0.5 or more were considered significantly associated with the factor. Only one factor proved to be significantly associated with most of the variables. The level of significance for the present study was set at 0.05.

Ethical considerations

This study was conducted according to the guidelines of the Declaration of Helsinki and approved by the Institutional Review Board of Ibn Sina National College for Medical Studies (approval number: H-07-09062021; approval date: 09.06.2021).

## Results

The present study included 286 participants (53.1% males and 46.9% females) to assess the knowledge of the population about AD using the Polish ADKS questionnaire [19]. The 25 questions of ADKS about the seven domains on clinical aspects and management of AD used in the present study were highly reliable, with a Cronbach’s alpha of 0.911. The percentage of correct answers is presented in Table 1. The majority of the correct responses for questions on the different subdomains were less than average. Responses are presented in Table 2.

**Table 1:** Frequencies of variables.

Variable	Category	Number frequency	Percentage
Gender	Male	152	53.1
	Female	134	46.9
	Total	286	100.0
Age (years)	Below 18	12	4.2
	18–25	89	31.1
	26–35	81	28.3
	36–45	52	18.2
	Above 45	52	18.2
	Total	286	100.0
Nationality	Saudi	247	86.4
	Non-Saudi	39	13.6
	Total	286	100.0
Family history of Alzheimer’s disease	No	239	83.6
	Parent	11	3.8
	Grandparents	36	12.6
	Total	286	100.0
Occupation	Unemployed	150	52.4
	Manual worker	61	21.3
	Clerical worker	75	26.2
	Total	286	100.0
Educational level	High school	64	22.4
	Diploma	43	15.0
	University	162	56.6
	Higher educational level	17	5.9
	Total	286	100.0
Smoking habit	Non-smoker	221	77.3
	Smoker	52	18.2
	Ex-smoker	13	4.5
	Total	286	100.0

**Table 2:** Distribution of the participants according to the correct answers to the questions on the different domains of Alzheimer’s disease (AD).

Question for each subdomain	Correct answers (%)
Life impact	
People with AD are particularly prone to depression	41.3%
Most people with AD live in nursing homes	10.8%
It is safe for people with AD to drive as long as they have a companion in the car at all times	23.1%
Risk factors	
It has been scientifically proven that mental exercises can prevent a person from getting AD	40.2%
People in their 30s can have AD	43.4%
Having high cholesterol may increase a person’s risk of developing AD	65.4%
Prescription drugs that prevent AD are available	52.4%
Having high blood pressure may increase a person’s risk of developing AD	56.6%
Genes can only partially account for the development of AD	39.5%
Course	
In rare cases, people have recovered from AD	36.4%
Eventually, a person with AD will need 24-hour supervision	17.1%
Assessment and diagnosis	
If trouble with memory and confused thinking appears suddenly, it is likely due to AD	26.2%
When a person with AD becomes agitated, a medical examination might reveal other health problems that caused the agitation	54.9%
Symptoms of severe depression can be mistaken for symptoms of AD	36.4%
AD is one type of dementia	20.3%
Treatment and management	
Poor nutrition can make the symptoms of AD worse	68.9%
AD cannot be cured	43.0%
Symptoms	
Tremor or shaking of the hands or arms is a common symptom in people with AD	43.0%
Trouble handling money or paying bills is a common early symptom of AD	31.8%
A symptom that can occur with AD is believing that other people are stealing one’s things	42.3%
Most people with AD remember recent events better than things that happened in the past	35.0%
Caregiving	
When people with AD repeat the same question or story several times, it is helpful to remind them that they are repeating themselves	20.6%
Once people have AD, they are no longer capable of making informed decisions about their own care	28.3%
When a person has AD, using reminder notes is a crutch that can contribute to the decline	37.4%
People with AD do best with simple instructions given one step at a time	51.0%

The maximum score for each subdomain and the mean score for the different categories of the sociodemographic variables are displayed in Table 3 and Table 4. Slight variations in the mean scores of the different categories of the sociodemographic variables were noticed. In general, the mean scores for the knowledge of the population on the subdomains of AD were substandard.

**Table 3:** Distribution of the participants according to sociodemographic characteristics and Alzheimer’s disease-related domain scores.

Variables		Risk factor		Domain symptoms		Domain course	
		Maximum score = 18		Maximum score = 12		Maximum score = 6	
	Categories	P	Mean	P	Mean	P	Mean
Age (years)	Below 18	0.922	14.7	0.373	10	0.358	4.8
	18–25		14.6		9.2		4.6
	26–35		14.7		9.5		4.7
	36–45		14.4		9.4		4.5
	Above 45		14.7		9.3		4.7
Education level	High school	0.010	15.1	0.075	9.7	0.270	4.7
	Diploma		15.0		9.5		4.8
	University		14.3		9.2		4.6
	Higher		14.2		9.1		4.6
Family history	No	0.350	14.6	0.057	9.4	0.045	4.6
	Parent		13.9		8.5		4.1
	Grandparents		14.8		9.5		4.6
Occupation	Unemployed	0.051	14.4	0.130	9.3	0.127	4.5
	Manual worker		15.1		9.7		4.8
	Clerical worker		14.5		9.3		4.7
Nationality	Saudi	0.566	14.6	0.403	9.3	0.518	4.6
	Non-Saudi		14.3		9.5		4.7
Gender	Male	0.018	14.6	0.140	9.3	0.031	4.6
	Female		14.5		9.3		4.6
Smoking	Non-smoker	0.291	14.4	0.504	9.3	0.673	4.6
	Smoker		14.6		9.3		4.7
	Ex-smoker		15.8		10.2		5.0

**Table 4:** Distribution of participants according to sociodemographic characteristics and Alzheimer’s disease-related domains.

Variables		Life impact		Caregiving		Assessment and diagnosis		Treatment and management	
		Maximum score = 12		Maximum score = 6		Maximum score = 12		Maximum score = 9	
	Categories	P	Mean	P	Mean	P	Mean	P	Mean
Age (years)	Below 18	0.515	9.7	0.819	4.3	0.698	10	0.174	7
	18–25		9.5		4.3		9.6		7
	26–35		9.7		4.3		9.7		7.4
	36–45		9.3		4.2		9.8		7.1
	Above 45		9.6		4.2		9.8		7.2
Education level	High school	0.029	9.8	0.141	4.4	0.252	9.9	0.362	7.2
	Diploma		9.7		4.4		9.8		7.4
	University		9.3		4.2		9.6		7.1
	Higher		9.7		4.3		9.7		7.5
Family history	No	0.067	9.5	0.215	4.3	0.572	9.7	0.528	7.2
	Parent		8.6		4		9.3		7.2
	Grandparents		9.6		4.5		9.7		7.4
Type of work	No	0.137	9.4	0.278	4.2	0.000	9.6	0.065	7.1
	Manual worker		9.8		4.4		10.3		7.5
	Clerical worker		9.5		4.3		9.4		7.9
Nationality	Saudi	0.484	9.5	0.968	4.3	0.339	9.7	0.868	7.2
	Non-Saudi		9.4		4.3		9.9		7.2
Gender	Male	0.034	9.7	0.000	4.3	0.029	9.6	0.041	7.2
	Female		9.4		4.1		9.8		7.2
Smoking	Non-smoker	0.756	9.4	0.000	4.2	0.047	9.6	0.474	7.2
	Smoker		9.6		4.4		9.8		7.2

The Pearson correlation coefficients for the scores on the seven subdomains of AD are presented in Table 5. High correlation coefficients were observed between the different scores, and these strong positive associations between the seven subdomains were also confirmed by the factor analysis where weights of the variables for the subdomains were significantly loaded on the same factor (factor 1) (Table 6).

**Table 5:** Correlation between all Alzheimer’s disease domains. *: P < 0.05; **: P < 0.01; ***: P < 0.001.

Subdomains		Risk factors	Symptoms	Course	Treatment and management	Assessment and diagnosis	Caregiving	Life impact
Risk factors	r	1						
Symptoms	r	0.687***	1					
Course	r	0.537***	0.563***	1				
Treatment and management	r	0.599***	0.490***	0.441***	1 -			
Assessment and diagnosis	r	0.642***	0.674***	0.497***	0.476***	1		
Caregiving	r	0.468***	0.567***	0.492***	0.372***	0.522***	1	
Life impact	r	0.696***	0.675***	0.545***	0.509***	0.585***	0.534***	1

**Table 6:** Principal component factor analysis for the knowledge, attitudes, and practice of the population toward Alzheimer’s disease (AD).

Variables	Weight of variables on Factor 1
People with AD are particularly prone to depression	0.502
It has been scientifically proven that mental exercises can prevent a person from getting AD	0.127
When a person with AD becomes agitated, a medical examination might reveal other health problems that caused the agitation	0.224
People with AD do best with simple instructions given one step at a time	0.530
In rare cases, people have recovered from AD	0.552
If trouble with memory and confused thinking appear suddenly, it is likely due to AD	0.652
Most people with AD live in nursing homes	0.588
Poor nutrition can make the symptoms of AD worse	0.516
People in their 30s can have AD	0.108
When people with AD repeat the same question or story several times, it is helpful to remind them that they are repeating themselves	0.662
Once people have AD, they are no longer capable of making informed decisions about their own care	0.635
Eventually, a person with AD will need 24-hour supervision	0.622
Having high cholesterol may increase a person’s risk of developing AD	0.520
Tremor or shaking of the hands or arms is a common symptom in people with AD	0.597
Symptoms of severe depression can be mistaken for symptoms of AD	0.654
AD is one type of dementia	0.531
Trouble handling money or paying bills is a common early symptom of AD	0.695
A symptom of AD is believing that other people are stealing one’s things	0.627
When a person has AD, using reminder notes is a crutch that can contribute to the decline	0.558
Prescription drugs that prevent AD are available	0.534
Having high blood pressure may increase a person’s risk of developing AD	0.558
Genes can only partially account for the development of AD	0.612
It is safe for people with AD to drive, as long as they have a companion in the car at all times	0.602
AD cannot be cured	0.604
Most people with AD remember recent events better than things that happened in the past	0.535

## Discussion

This research is among the first investigations of the population’s knowledge in Saudi Arabia regarding AD based on the Polish ADKS. This study confirmed the findings of previous studies on the validity and reliability of this questionnaire [19-21]. In the present study, all questions of the questionnaire were significantly associated, as proven by the factor analysis and Cronbach’s alpha of 0.911. Significant associations between the seven subdomains were found in this study. Life impact was found to be associated with caregiving, symptoms, and treatment. Moreover, risk factors, assessment and diagnosis, caregiving, and management and treatment were found to be associated with the course of AD. As the intervention for AD is multidisciplinary, it is worth noting that the well-being of the patient is directly influenced by these factors [22]. Increased educational levels had lower scores on the subdomains of risk factors and life impact. This is contradictory to the findings of other studies [23-25]. Female participants had less retention of knowledge on life impact, caregiving, treatment and management, risk factors, and course than males. On the other hand, males had higher scores on the subdomains of assessment and diagnosis. This is not in line with other studies [26,27]. Previous studies found that advancing age and postgraduate training were significantly associated with better information on knowledge about AD [26,28]. However, in this study, we did not find such an association. Meanwhile, it was shown in the present study that higher knowledge on the course subdomain was associated with having relatives diagnosed with AD, which could be related to gaining knowledge through their experience with their affected relatives. Previous studies found similar associations [5,18]. The factor analysis employed in this study revealed that all elements of the seven subdomains of knowledge score about AD were significantly related; thus, identifying the knowledge gap is crucial to bridge the gap in knowledge of the public about AD. Symptoms were found to be associated with treatment and management. As AD is managed by multiple specialties, integration of these subdomains is essential for the well-being of the patient [29]. Lack of knowledge on caregiving may to some extent delay the management and interventions provided to patients diagnosed with AD and lead to adverse effects on their quality of life [30].

Limitations

There are some limitations of this study. As this study was cross-sectional, and the sampling technique was a non-probability convenient one, its generalization to the population may be limited; however, this study was an exploratory investigation.

## Conclusions

In this study, the majority of studied participants had a lower level of knowledge on all domains of knowledge about AD. All subdomains of knowledge about AD were significantly correlated and potentiated each other. Upgrading the knowledge of the public about these subdomains will improve the management of AD, with subsequent improvement in the quality of life of affected individuals. Thus, national awareness campaigns for all parts of the community are essential to enhance awareness about AD.

## Appendices

**Table 7:** Questionnaire items.

1	People with Alzheimer’s disease are particularly prone to depression	True	False
2	It has been scientifically proven that mental exercise can prevent a person from getting Alzheimer’s disease	True	False
3	After symptoms of Alzheimer’s disease appear, the average life expectancy is 6 to 12 years	True	False
4	When a person with Alzheimer’s disease becomes agitated, a medical examination might reveal other health problems that caused the agitation	True	False
5	People with Alzheimer’s disease do best with simple, instructions given one step at a time.	True	False
6	When people with Alzheimer’s disease begin to have difficulty taking care of themselves, caregivers should take over right away	True	False
7	If a person with Alzheimer’s disease becomes alert and agitated at night, a good strategy is to try to make sure that the person gets plenty of physical activity during the day.	True	False
8	In rare cases, people have recovered from Alzheimer’s disease	True	False
9	People whose Alzheimer’s disease is not yet severe can benefit from psychotherapy for depression and anxiety	True	False
10	If trouble with memory and confused thinking appears suddenly, it is likely due to Alzheimer’s disease	True	False
11	Most people with Alzheimer’s disease live in nursing homes	True	False
12	Poor nutrition can make the symptoms of Alzheimer’s disease worse	True	False
13	People in their 30s can have Alzheimer’s disease	True	False
14	A person with Alzheimer’s disease becomes increasingly likely to fall as the disease gets worse	True	False
15	When people with Alzheimer’s disease repeat the same question or story several times, it is helpful to remind them that they are repeating themselves	True	False
16	Once people have Alzheimer’s disease, they are no longer capable of making informed decisions about their own care	True	False
17	Eventually, a person with Alzheimer’s disease will need 24-hour supervision	True	False
18	Having high cholesterol may increase a person’s risk of developing Alzheimer’s disease	True	False
19	Tremor or shaking of the hands or arms is a common symptom in people with Alzheimer’s disease	True	False
20	Symptoms of severe depression can be mistaken for symptoms of Alzheimer’s disease	True	False
21	Alzheimer’s disease is one type of dementia	True	False
22	Trouble handling money or paying bills is a common early symptom of Alzheimer’s disease	True	False
23	One symptom that can occur with Alzheimer’s disease is believing that other people are stealing one’s things	True	False
24	When a person has Alzheimer’s disease using reminder notes is a crutch that can contribute to the decline	True	False
25	Prescription drugs that prevent Alzheimer’s disease are available	True	False
26	Having high blood pressure may increase a person’s risk of developing Alzheimer’s disease	True	False
27	Genes can only partially account for the development of Alzheimer’s disease	True	False
28	It is safe for people with Alzheimer’s disease to drive, as long as they have a companion in the car at all times	True	False
29	Alzheimer’s disease cannot be cured	True	False
30	Most people with Alzheimer’s disease remember recent events better than things that happened in the past.	True	False
